# Eosinophils affect functions of in vitro-activated human CD3-CD4+ T cells

**DOI:** 10.1186/1479-5876-11-112

**Published:** 2013-05-06

**Authors:** Issam Harfi, Liliane Schandené, Sarah Dremier, Florence Roufosse

**Affiliations:** 1 Institute for Medical Immunology, Université Libre de Bruxelles, Gosselies, Belgium; 2 Laboratory of Immunology, Hôpital Erasme, Université Libre de Bruxelles, Brussels, Belgium; 3 Department of Internal Medicine, Hôpital Erasme, Université Libre de Bruxelles, 808 Route de Lennik, Brussels, B-1070, Belgium

**Keywords:** Eosinophil-targeted therapy, CD4 T-cells, CD3^-^CD4^+^, Lymphocytic variant hypereosinophilic syndrome, Anti-IL-5

## Abstract

**Background:**

The recent development of eosinophil-targeting agents has raised enthusiasm for management of patients with hypereosinophilic syndromes. Roughly half of anti-IL-5-treated patients with corticosteroid-responsive lymphocytic (L-HES) and idiopathic disease variants can be tapered off corticosteroids. Potential consequences of corticosteroid-withdrawal on clonal expansion of pre-malignant CD3^-^CD4^+^ T-cells associated with L-HES are a subject of concern. Indeed, corticosteroid treatment inhibits T-cell activation and may lower blood CD3^-^CD4^+^ cell counts. On the other hand, previous studies have shown that eosinophils support CD4 T-cell activation, suggesting that targeted eosinophil depletion may negatively regulate these cells.

**Objectives:**

Effects of eosinophils on CD4 T-cell activation in vitro were investigated as an indirect means of exploring whether treatment-induced eosinophil depletion may affect pathogenic T-cells driving L-HES.

**Methods:**

Helper (CD4) T-cells and CD3^-^CD4^+^ cells from healthy controls and L-HES patients, respectively, were cultured in vitro in presence of anti-CD3/CD28 or dendritic cells. Effects of eosinophils on T-cell proliferation and cytokine production were investigated.

**Results:**

Eosinophils enhanced CD3-driven proliferation of CD4 T-cells from healthy subjects in vitro, while inhibiting TCR-independent proliferation and IL-5 production by CD3^-^CD4^+^ T-cells.

**Conclusions:**

While this study confirms previous work showing that eosinophils support activation of normal helper T-cells, our in vitro findings with CD3^-^CD4^+^ T-cells suggest that eosinophil-depletion may favor activation and expansion of this pathogenic lymphocyte subset. With the ongoing development of eosinophil-targeted therapy for various eosinophilic conditions, the indirect consequences of treatment on the underlying immune mechanisms of disease should be investigated in detail in the setting of translational research programs.

## Introduction

Hypereosinophilic syndromes (HES) are characterized by eosinophil-mediated tissue and organ damage, occurring in the setting of persistent hypereosinophilia [1]. Although several HES variants have been defined on the basis of pathogenic disease mechanisms, the majority of patients are still classified as “idiopathic” HES. In lymphocytic variant HES (L-HES), hypereosinophilia develops in response to marked over-production of eosinophilopoietic cytokines [most notably interleukin (IL)-5] by clonally expanded T-cells that often bear a CD3^-^CD4^+^ phenotype [2]. A small subset of patients with L-HES may develop T-cell lymphoma years after diagnosis, possibly in relation with occurrence of specific cytogenetic abnormalities [3].

For patients with both idiopathic HES and L-HES, corticosteroids (CS) represent first-line therapy [4]. Interferon (IFN)-α is an alternative for those who are CS-dependent or CS-resistant. Both treatment options target T-cell functions and have the potential to reduce CD3^-^CD4^+^ T-cell counts [2]. Treatment of HES is not curative and must therefore be maintained long-term to prevent the development of irreversible eosinophil-mediated organ damage. As a result, significant treatment-related toxicity and development of drug-resistance are common.

The recent development of targeted eosinophil-specific therapies has aroused enthusiasm for HES management. Interleukin-5 is a key mediator involved in differentiation, expansion, chemotaxis, activation, and survival of eosinophils. Because of restricted expression of the human IL-5 receptor α chain (IL-5Rα), monoclonal antibodies directed against IL-5 or its receptor are expected to have a selective impact on eosinophils [5]. The anti-IL-5 antibody, mepolizumab, was recently shown to be an effective CS-sparing and eosinophil-lowering agent in CS-responsive HES patients with both lymphocytic and idiopathic disease variants [6-8]. Roughly half of mepolizumab-treated patients can durably be tapered off CS. Although this represents a major step forward for patients with HES, withdrawal of therapeutic agent(s) active on T-cells is a subject of concern for patients with L-HES because of the pre-malignant potential of CD3^-^CD4^+^ T-cells.

The perspective of eosinophil-targeted therapy for HES has therefore raised new questions, one of which pertains to the fate of pathogenic CD4 T-cells. The present study was undertaken to explore this issue, with a special focus on clonal CD3^-^CD4^+^ T-cells associated with L-HES. The effects of eosinophils on CD4 T-cell activation were investigated in vitro as an indirect means of exploring potential consequences of treatment-induced eosinophil depletion.

## Material and methods

### Patients and control subjects

For the in vitro co-culture studies, fresh blood was drawn from healthy controls recruited within our institution for isolation of eosinophils and CD4 T-cells, and generation of monocyte-derived dendritic cells (DC). For experiments conducted on CD3^-^CD4^+^ T-cells, we used a stock of peripheral blood mononuclear cells (PBMC) previously obtained by cytapheresis from two patients with L-HES (patients L-HES_1,2_), and stored in liquid nitrogen. The cells from these two patients were considered fully representative of L-HES-associated CD3^-^CD4^+^ T-cells, which have been shown to express an extremely homogenous phenotype [9] and gene expression profile [10]. The clinical history of one patient (L-HES_1_) has been described previously (patient 3 in reference [9]); she was off treatment at the time cytapheresis was performed. Patient L-HES_2_ had interrupted corticosteroid monotherapy 48 hours prior to cytapheresis. All subjects provided written consent, and this study was approved by our Institutional Review Board.

### Isolation of eosinophils and CD4 T-cells

Blood from healthy donors was subjected to density gradient centrifugation using Lymphoprep (Lucron, ELITechGroup, Belgium). PBMCs were harvested and left at room temperature overnight in RPMI (Lonza, Verviers, Belgium) containing 10% fetal calf serum, Penstrep, and glutamine (all from Life Technologies Europe, Gent, Belgium) (RPMI-FCS), before purification of CD4 T-cells. The remaining cells were subjected to red-blood-cell lysis on ice using NH_4_Cl 0.8%. Granulocytes were re-suspended in PBS (Lonza) with EDTA (2mM) and BSA (0.5%) (Sigma-Aldrich, St. Louis, MO), and eosinophil isolation was performed at 4°C using a human Eosinophil Isolation Kit (Miltenyi Biotec, Bergisch Gladbach, Germany) according to manufacturer’s instructions (negative selection). Viability and degree of purification of eosinophils were >95% as assessed by Trypan-Blue and May-Grünwald-Giemsa staining. Eosinophils were resuspended in RPMI-FCS at 2×10^6^ cells per ml, and activated in vitro for 18 hours (37°C, 5% CO_2_) prior to co-incubation with CD4 T-cells, using human IgA (7,5 μg/ml)(Sigma-Aldrich, human IgA from colostrum), and affinity-purified goat anti-human IgA (10 μg/ml) (Gentaur). This strategy was chosen to elicit eosinophil activation prior to co-incubation with T-cells, thereby optimizing their costimulatory properties.

Purified CD4 T-cells (healthy donors and L-HES patients) were obtained from PBMC using the human CD4^+^ T cell Isolation Kit II (Miltenyi Biotec) according to manufacturer’s instructions (negative selection). CD4 T-cells from patients L-HES_1,2_ were further subjected to a Dynabeads CD3 kit (Invitrogen, Life Technologies) to obtain purified CD3^-^CD4^+^ T cells. Purity of desired cells was >95%.

### Preparation of monocyte-derived dendritic cells

Monocytes were purified from healthy subject PBMCs using the human CD14 MicroBeads kit (Miltenyi Biotec) according to manufacturer’s instructions (positive selection). CD14-positive cells were resuspended in RPMI-FCS enriched with non-essential amino acids (NEAA, Lonza Biowhittaker) (RPMI-FCS-NEAA) at 5×10^5^/per ml, cultured for 6 days at 37°C, 5% CO2, and fed twice with GM-CSF (800 U/ml) and IL-4 (500 U/ml) (Gentaur, Kampenhout Belgium). On day-6, LPS (100 ng/ml) (Sigma-Aldrich) was added. DCs harvested on day-7 expressed a mature phenotype as assessed by flow cytometry (high expression of CD80, CD86, CD83, HLA-DR).

### Co-culture experiments

For healthy control co-culture experiments, eosinophils and T-cells were autologous, and T-cells were activated with pre-coated anti-hCD3ϵ (10 μg/ml in PBS, 30 μl per well, overnight, 4°C) (R&D Systems, Minneapolis) and soluble anti-CD28 (1 μg/ml) (Immunotech, clone 28.2). For experiments using CD3^-^CD4^+^ cells (L-HES), we could not use combined anti-CD3/CD28 antibodies to induce activation, as these cells do not respond to CD3 engagement. They do, however, respond to costimulatory signals (CD2 and CD28) provided by monocyte-derived LPS-matured DC (i.e. TCR-independent activation), as demonstrated previously [11]. For the present study, CD3^-^CD4^+^ T-cells were therefore activated with monocyte-derived DC from healthy donors, and then co-incubated with eosinophils from the same donor.

Co-cultures were performed in 96-well flat-bottom culture plates (Greiner Bio-one, Cellstar). For healthy controls, CD4 T-cells (10^5^ cells per well) and eosinophils (2×10^5^ cells per well for a 2/1 ratio, 10^5^ cells for a 1/1 ratio, 5×10^4^ for a 0.5/1 ratio, and 10^4^ cells for a 0.1/1 ratio) were co-cultured for 48 hours at 37°C, 5% CO_2_ in RPMI-FCS. For L-HES co-cultures, CD3^-^CD4^+^ T-cells (10^5^ cells per well), LPS-matured DC (10^4^ cells per well), and eosinophils (2×10^5^ cells per well) were cultured for 5 days at 37°C, 5% CO_2_ in RPMI-FCS-NEAA.

At the end of co-cultures, 1/ culture supernatants were harvested and stored at −20°C for cytokine measurements (ELISA), 2/ cells were assessed for membrane antigen expression, and/or intracellular cytokine expression, and 3/ H^3^-thymidine was added (triplicate) for 18 hours to quantify proliferation.

### Quantification of membrane-expressed antigens and cytokine expression

Freshly thawed PBMC from L-HES patients and from 3 healthy controls were used to quantify expression of IL-5Rα. Cells were stained with anti-CD3-FITC, anti-CD4-PerCP and anti-CD125w-PE or the relevant isotype control.

Flow cytometry was also used at the end of co-cultures for assessment of membrane antigen expression and intracellular cytokine expression by T-cells. All cell types (T-cells, eosinophils, and DC) were readily distinguished on the basis of their forward and side-scatter properties. A large population of viable eosinophils with high side-scatter (i.e. granularity) was clearly identified at the end of all co-cultures. For surface antigen assessment, T-cells were directly labeled with fluorochrome-conjugated antibodies at the end of co-cultures (anti-CD3-FITC, anti-CD4-PerCP, anti-HLA-DR-PE, anti-CD25-APC). For intracellular cytokine quantification, cells were incubated with Brefeldine A (10 μg/ml), phorbol 12-myristate 13-acetate (PMA) (50 ng/ml) and the ionophore A23187 (100 ng/ml) (all from Sigma) for 4 additional hours, stained with anti-CD3-FITC and anti-CD4-PerCP, fixed and permeabilized using the Caltag cell permeabilisation kit (Life Technologies), then incubated with anti-IL-2-PE and anti-IL-5-APC. Successful activation of T-cells was confirmed by demonstrating increased CD69 expression in stimulated compared to unstimulated cells. Acquisition was performed using a FacsCanto II with Diva software (Becton Dickinson, San José, CA) (gate on living cells). Antibodies were purchased from BD/BD-Pharmingen.

Co-culture supernatants were thawed for cytokine measurements using commercial kits for IL-5 (R&D, Quantikine) and IL-2 (eBioscience, ready-set-go), used according to manufacturer’s instructions.

### Statistical analysis

All paired observations (proliferation, CD25 and IL-2 expression by CD4 T-cells in absence versus in presence of eosinophils) were subjected to Wilcoxon signed rank test. For co-culture experiments with DC, results for the 3 conditions (T-cells versus T-cells + DC versus T-cells + DC + eosinophils) were analysed with Kruskal-Wallis and compared two-by-two using Wilcoxon rank sum exact test.

## Results and discussion

**Figure 1:**
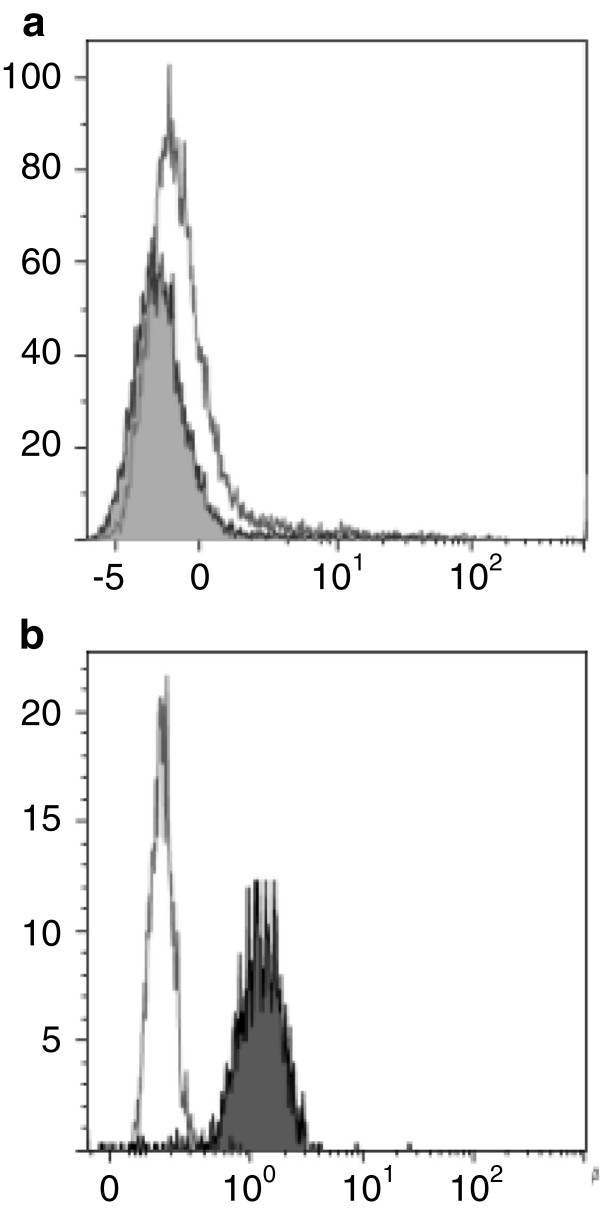
**L-HES-associated CD3**^**-**^**CD4**^**+ **^**T-cells do not express IL-5Rα.** CD3^-^CD4^+^ T-cells from patient L-HES_1_ stain negatively for CD125w (grey histogram), similar to normal CD4 T-cells (white histogram) (1**a**), whereas basophils stain positively for this receptor (grey histogram, 1**b**), as expected. Identical results were obtained for CD3^-^CD4^+^ T-cells from patient L-HES_2_ and a third patient with L-HES.

Eosinophil-specific therapeutic agents targeting IL-5 or its receptor would not be expected to directly affect CD4 T-cells, which are known not to express the IL-5 receptor [12]. We first established that this is also true for CD3^-^CD4^+^ T-cells, showing that aberrant cells from 3 patients with L-HES stain negatively for the IL-5Rα chain (Figure 1).

**Figure 2:**
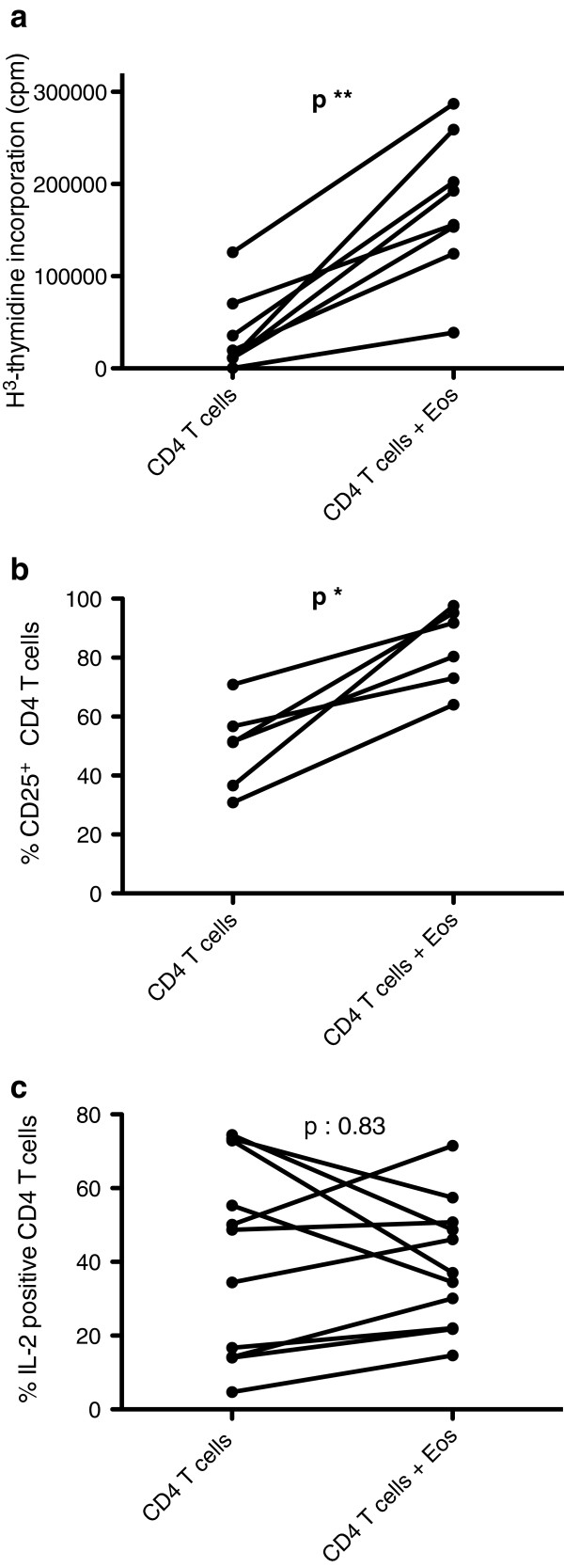
**Eosinophils enhance proliferation and CD25 expression of in vitro activated CD4 T-cells from healthy controls.** Purified CD4^+^ T-cells from healthy subjects were activated with coated anti-CD3 and soluble anti-CD28 (1 μg/ml) antibodies for 48 hours, in absence and in presence of autologous IgA/anti-IgA activated eosinophils. Cells were then cultured for an additional 18 hours with H^3^-thymidine to assess proliferation (n=8) (2**a**), washed and stained with fluoro-conjugated antibodies for assessment of membrane CD25 expression by flow cytometry (n=6) (2**b**), or underwent brief re-stimulation with PMA and A23187 to assess intracellular cytokine expression (n=10) (2**c**).

Therapeutic eosinophil depletion may however affect CD4 T-cells indirectly, by interrupting cross-talk between these two cell-types. To explore this eventuality, we first investigated whether eosinophils affect in vitro activation of CD4 T-cells from healthy subjects. CD4 T-cells were cultured in presence of anti-CD3/CD28 antibodies for 48 hours, in absence and in presence of autologous IgA/anti-IgA pre-activated eosinophils. Eosinophils consistently and significantly increased T-cell proliferation (Figure 2a), as assessed by H^3^-thmidine incorporation (median 15722 versus 174332 cpm, in absence and in presence of eosinophils respectively, p = 0.008). This effect was dose-dependent, with proliferation increasing in parallel with eosinophil-T cell ratios (Additional file 1: Figure S1). Eosinophils also further increased membrane CD25 expression by CD4 T-cells (Figure 2b) (median proportion of CD25^+^ CD4 T-cells 51.4 versus 86.1% in absence and in presence of eosinophils respectively, p = 0.03). In contrast, we did not observe a statistically significant effect on the proportion of IL-2 (Figure 2c), or IL-5 (data not shown)-expressing T-cells as assessed by flow cytometry, and measurement of IL-2 in culture supernatants did not yield reproducible results (not shown).

**Figure 3:**
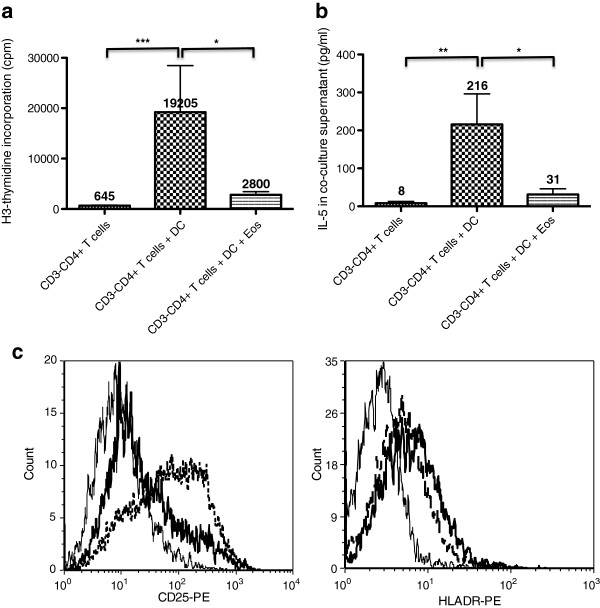
**Eosinophils inhibit dendritic cell-induced activation of CD3**^**-**^**CD4**^**+ **^**T cells in vitro.** Purified CD3^-^CD4^+^ T cells were cultured in presence of LPS-matured dendritic cells from healthy subjects for 5 days, in absence or in presence of IgA/anti-IgA-activated eosinophils. Proliferation was assessed on the basis of H^3^-thymidine incorporation (3**a**), IL-5 was measured in culture supernatants by ELISA (3**b**), and expression of activation markers CD25 and HLA-DR was assessed by flow cytometry (3**c**). Histograms represent mean proliferation (cpm) (3a, n=8) and IL-5 concentrations (pg/ml) (3b, n=6), and bars show the standard error of the mean for each condition. For cytometry studies (3**c**), histograms show CD25 and HLA-DR expression on CD3^-^CD4^+^ T cells cultured alone (continuous fine line), with dendritic cells (dashed bold line), and with both dendritic cells and eosinophils (continuous bold line); results are representative of 3 independent experiments.

To more specifically address the fate of clonal T-cells in L-HES patients treated with eosinophil-depleting therapy, we set up cultures with CD3^-^CD4^+^ T-cells from L-HES patients, in absence and in presence of eosinophils. In these experiments, T-cell activation was achieved using allogeneic mature DC, which provide costimulatory, TCR-independent signals to CD3^-^CD4^+^ cells [11]. Consistent with our previous work, DC induced proliferation of CD3^-^CD4^+^ cells (Figure 3a) and secretion of IL-5 (Figure 3b). Eosinophils consistently and significantly reduced DC-induced proliferation of the aberrant T-cells (median reduction 58%), as well as IL-5 production (median reduction 93%). We previously reported that DC-induced CD3^-^CD4^+^ T-cell proliferation and Th2 cytokine production involve IL-2/IL-2Rα interactions [11]. We did not observe any reproducible eosinophil-induced changes in IL-2 concentrations in culture supernatants (not shown), which were low or undetectable in all culture conditions, presumably due to recapture by activated T-cells. Attempts to re-stimulate the cells at the end of co-cultures for detection of intracytoplasmic cytokine expression were unrewarding in the majority of experiments; however, we did observe a clear-cut decrease in the percentage of IL-2-positive and IL-5-positive T-cells from patient L-HES_1_ when eosinophils were added (Additional file 2: Figure S2). As for IL-2Rα, CD3^-^CD4^+^ T-cells from patient L-HES_1_ showed increased membrane expression in response to DC, and eosinophils consistently lowered the percentage of positively stained cells (Figure 3c).

Overall, these in vitro experiments suggest that eosinophils display differential effects on healthy activated CD4 T-cells, and on abnormal clonal CD3^-^CD4^+^ T-cells found in L-HES.

The data presented herein with normal CD3/CD28-activated CD4 T-cells, supports previous studies indicating that eosinophils potentiate CD3-mediated T-cell activation. Eosinophils have indeed been shown to support antigen and super-antigen induced activation and proliferation of normal CD4 T-cells in a HLA-DR dependent fashion [13-15]. It is therefore conceivable that eosinophil depletion in clinical situations typically associated with antigen-driven T-cell activation and (hyper)eosinophilia, including allergic disorders, may ultimately blunt these T-cell responses to some degree in vivo. Our observation that CD4 T cell proliferation progressively decreases in parallel with eosinophil concentrations indicates that tighter therapeutic control of eosinophil levels in patients with reactive eosinophilia may result in inhibition of other down-stream consequences of pathogenic T cell activation.

The relevance of these observations for humans receiving eosinophil-targeted therapy remains elusive. Others have explored short-term (maximum 3 monthly infusions) effects of anti-IL-5 treatment on normal CD4 T-cells in vivo, in patients with asthma, idiopathic HES, and eosinophilic esophagitis [16-18]. Although these studies suggest overall that therapeutic eosinophil depletion has no significant effects on the biology of normal CD4 T-cells, the observation period is short compared to the prolonged requirement for treatment in patients with allergic disorders and HES. Long-term in vivo evaluation of CD4 T-cell biology in patients treated with eosinophil-depleting therapy, together with longitudinal in vitro quantification of pertinent antigen-specific responses are warranted.

In contrast to normal CD4 T-cells, CD3^-^CD4^+^ Th2-cell functions were consistently inhibited in vitro by eosinophils in the present study. This may be related to intrinsic properties of the CD3^-^CD4^+^ T-cells and/or their reliance on TCR/CD3-independent, rather than TCR-dependent, activation pathways. Indeed, studies reported so far have shown a central role of HLA-DR, and thus TCR engagement, in eosinophil-mediated activation of T-cells [13-15]. Because L-HES-associated CD3^-^CD4^+^ cells lack membrane TCR/CD3 expression, they are unable to interact with MHC molecules, and our recent study on their gene expression profile has shown reduced expression of numerous CD3-related intracellular signaling molecules [10]. In contrast, these cells have been shown to respond to costimulatory ligands [11], some of which are expressed by eosinophils [14,19]. Eosinophil-derived signals may therefore affect downstream signaling pathways differently, depending on whether the T-cells are activated through the TCR/CD3 complex or not. Besides reduced expression of many CD3-associated genes, significant alterations in the expression level of various other membrane-expressed receptors and signaling pathways were observed in CD3^-^CD4^+^ cells [10], some of which may be relevant with regard to interactions with eosinophils.

Our findings suggest that eosinophils may actually play a regulatory role on the global activation status of CD3^-^CD4^+^ T-cells. Should this be the case in vivo, eosinophil depletion with eosinophil-targeted treatment may actually favor clonal T-cell outgrowth in some patients with L-HES. Several patients with L-HES have been treated with anti-IL-5 for several years in the setting of an open-label study, but none have been reported to develop T-cell lymphoma [8]. Although two patients with idiopathic HES enrolled in the study did develop lymphoma during the observation period, neither of them had detectable CD3^-^CD4^+^ T-cells. Long-term, longitudinal, in vivo data on CD4 T-cells in patients with L-HES receiving eosinophil-targeted therapy is currently unavailable. Absolute counts of abnormal T-cells in patients with L-HES were not systematically assessed throughout the above-mentioned open-label study to assess for clonal expansion, nor were TCR gene rearrangement analyses performed on the entire cohort to check for appearance of new T-cell clones. Our in vitro data emphasize the need for punctilious in vivo assessment of aberrant T-cell clones in the setting of long-term eosinophil-targeting treatment protocols. Although the risk of developing lymphoma in patients with L-HES is low, careful clinical follow-up is warranted, regardless of the chosen treatment strategy.

In conclusion, our data provide further evidence that activated eosinophils interact directly with CD4 T-cells and may affect their functions in diseases associated with (hyper)eosinophilia. Additionally, in vivo, tissue-dwelling eosinophils have been shown to interact with numerous other immune and non-immune cell types thereby indirectly affecting T cell functions [20]. With increasing opportunities to treat various eosinophilic conditions using eosinophil-targeted therapy, special attention should be paid to potential consequences of eosinophil depletion on T-cell biology. Specifically, the present findings suggest that eosinophils may restrain expansion of clonal CD3^-^CD4^+^ T-cells associated with L-HES, perhaps contributing to the indolent nature of this pre-malignant disease. Future investigations should focus on the eosinophil-derived mediators and surface molecules affecting CD3^-^CD4^+^ T-cell proliferation and activation.

## Abbreviations

APC: Allophycocyanin; BSA: Bovine serum albumine; CS: Corticosteroid; DC: Dendritic cell; EDTA: Ethylenediaminetetraacetic acid; ELISA: Enzyme linked immuno-sorbent assay; FCS: Fetal calf serum; FITC: Fluorescein; GM-CSF: Granulocyte monocyte colony stimulating factor; HES: Hypereosinophilic syndrome; HLA: Human leukocyte antigen; IFN: Interferon; IL-: Interleukin; IL-2Rα: IL-2 receptor alpha; IL-5Rα: IL-5 receptor alpha; L-HES: Lymphocytic variant HES; LPS: Lipopolysaccharide; MDC: Macrophage derived chemokine; MHC: Major histocompatibility complex; NEAA: Non essential amino acid solution; PBMC: Peripheral blood mononuclear cells; PBS: Phosphate buffered saline; PE: Phycoerythrin; PerCP: Peridinin chlorphyll protein; PMA: Phorbol 12-myristate 13-acetate; RPMI: Roswell Park Memorial Institute (medium); TARC: Thymus and activation regulated chemokine; TCR: T cell receptor; TH2: Type 2 helper T cells.

## Competing interests

FR has punctually received a consultancy fee from GlaxoSmithKline. The authors declare that they have no competing interests.

## Authors’ contributions

IH designed the study, determined optimal culture conditions, performed most experiments reported herein, and drafted the manuscript. SD performed and interpreted the preparatory experiments establishing successful eosinophil activation in vitro. LS performed and interpreted cytometry studies. FR conceived the study, participated in its design, performed many experiments, interpreted the data, performed statistical analysis, and drafted the manuscript. All authors read and approved the manuscript.

## Supplementary Material

Additional file 1: Figure S1 Eosinophil-induced enhancement of CD4 T cell proliferation is dose-dependent. Purified CD4^+^ T-cells from healthy subjects were activated with coated anti-CD3 and soluble anti-CD28 (1 μg/ml) antibodies for 48 hours, in absence and in presence of autologous IgA/anti-IgA activated eosinophils at various eosinophil-T cell ratios (0.1/1, 0.5/1, 1/1, and 2/1). Cells were then cultured for an additional 18 hours with H^3^-thymidine to assess proliferation. A statistically highly significant dose–response curve was observed when comparing T-cells alone to T-cells cultured with eosinophils at eosinophil/T cell ratios at 0.5/1 and 2/1 (Additional file 1: Figure S1a, n=7). A statistically significant progression was also observed when exploring additional ratios (Additional file 1: Figure S1b, n=4). Click here for file

Additional file 2: Figure S2 Eosinophils decrease the percentage of CD3^-^CD4^+^ T-cells expressing IL-2 and IL-5. CD3^-^CD4^+^ T-cells from patient L-HES_1_ were cultured alone, in presence of LPS-matured dendritic cells, or in presence of both LPS-matured dendritic cells and eosinophils. On day 5, cells were harvested, washed, and re-stimulated with PMA+A23187 in presence of brefeldine A for a further 4 hours. Cells were then surface-stained with anti-CD3-FITC and anti-CD4-PerCP, fixed and permeabilised, then stained for intracytoplasmic cytokine expression with anti-IL-2-PE and anti-IL-5-APC. Acquisition for flow cytometry was performed on a Facs Canto II, using DIVA software. Click here for file

## References

[B1] Valent P Klion AD Horny HP Roufosse F Gotlib J Weller PF Contemporary consensus proposal on criteria and classification of eosinophilic disorders and related syndromes J Allergy Clin Immunol 2012 130 3 607 612 10.1016/j.jaci.2012.02.019 22460074 PMC4091810

[B2] Roufosse F Cogan E Goldman M Lymphocytic variant hypereosinophilic syndromes Immunol Allergy Clin North Am 2007 27 3 389 413 10.1016/j.iac.2007.07.002 17868856

[B3] Ravoet M Sibille C Roufosse F Duvillier H Sotiriou C Schandené L 6q- is an early and persistent chromosomal aberration in CD3-CD4+ T-cell clones associated with the lymphocytic variant of hypereosinophilic syndrome Haematologica 2005 90 6 753 765 15951288

[B4] Klion AD How I treat hypereosinophilic syndromes Blood 2009 114 18 3736 3741 10.1182/blood-2009-07-143552 19692700 PMC2773488

[B5] Molfino NA Gossage D Kolbeck R Parker JM Geba GP Molecular and clinical rationale for therapeutic targeting of interleukin-5 and its receptor Clin Exp Allergy 2012 42 5 712 737 10.1111/j.1365-2222.2011.03854.x 22092535

[B6] Rothenberg ME Klion AD Roufosse FE Kahn JE Weller PF Simon HU Treatment of patients with the hypereosinophilic syndrome with mepolizumab N Engl J Med 2008 358 12 1215 1228 10.1056/NEJMoa070812 18344568

[B7] Roufosse F de Lavareille A Schandene L Cogan E Georgelas A Wagner L Mepolizumab as a corticosteroid-sparing agent in lymphocytic variant hypereosinophilic syndrome J Allergy Clin Immunol 2010 126 4 828 835 e3 10.1016/j.jaci.2010.06.049 20810155 PMC2950246

[B8] Roufosse FE Kahn JE Gleich GJ Schwartz LB Singh AD Rosenwasser LJ Long-term safety of mepolizumab for the treatment of hypereosinophilic syndromes J Allergy Clin Immunol 2013 131 2 461 467 10.1016/j.jaci.2012.07.055 23040887 PMC3558744

[B9] Roufosse F Schandene L Sibille C Willard-Gallo K Kennes B Efira A Clonal Th2 lymphocytes in patients with the idiopathic hypereosinophilic syndrome Br J Haematol 2000 109 3 540 548 10.1046/j.1365-2141.2000.02097.x 10886202

[B10] Ravoet M Sibille C Gu C Libin M Haibe-Kains B Sotiriou C Molecular profiling of CD3-CD4+ T cells from patients with the lymphocytic variant of hypereosinophilic syndrome reveals targeting of growth control pathways Blood 2009 114 14 2969 2983 10.1182/blood-2008-08-175091 19608752

[B11] Roufosse F Schandene L Sibille C Kennes B Efira A Cogan E T-cell receptor-independent activation of clonal Th2 cells associated with chronic hypereosinophilia Blood 1999 94 3 994 1002 10419891

[B12] Wilson TM Maric I Shukla J Brown M Santos C Simakova O IL-5 receptor alpha levels in patients with marked eosinophilia or mastocytosis J Allergy Clin Immunol 2011 128 5 1086 1092 e1-3 10.1016/j.jaci.2011.05.032 21762978 PMC3205313

[B13] Weller PF Rand TH Barrett T Elovic A Wong DT Finberg RW Accessory cell function of human eosinophils. HLA-DR-dependent, MHC-restricted antigen-presentation and IL-1 alpha expression J Immunol 1993 150 6 2554 2562 8450230

[B14] Celestin J Rotschke O Falk K Ramesh N Jabara H Strominger J IL-3 induces B7.2 (CD86) expression and costimulatory activity in human eosinophils J Immunol 2001 167 11 6097 6104 11714768 10.4049/jimmunol.167.11.6097

[B15] Mawhorter SD Kazura JW Boom WH Human eosinophils as antigen-presenting cells: relative efficiency for superantigen- and antigen-induced CD4+ T-cell proliferation Immunology 1994 81 4 584 591 7518797 PMC1422364

[B16] Buttner C Lun A Splettstoesser T Kunkel G Renz H Monoclonal anti-interleukin-5 treatment suppresses eosinophil but not T-cell functions Eur Respir J 2003 21 5 799 803 10.1183/09031936.03.00027302 12765424

[B17] Plotz SG Simon HU Darsow U Simon D Vassina E Yousefi S Use of an anti-interleukin-5 antibody in the hypereosinophilic syndrome with eosinophilic dermatitis N Engl J Med 2003 349 24 2334 2339 10.1056/NEJMoa031261 14668459

[B18] Stein ML Villanueva JM Buckmeier BK Yamada Y Filipovich AH Assa'ad AH Anti-IL-5 (mepolizumab) therapy reduces eosinophil activation ex vivo and increases IL-5 and IL-5 receptor levels J Allergy Clin Immunol 2008 121 6 1473 1483 83 e1-4 10.1016/j.jaci.2008.02.033 18410960 PMC2749495

[B19] Woerly G Roger N Loiseau S Dombrowicz D Capron A Capron M Expression of CD28 and CD86 by human eosinophils and role in the secretion of type 1 cytokines (interleukin 2 and interferon gamma): inhibition by immunoglobulin a complexes J Exp Med 1999 190 4 487 495 10.1084/jem.190.4.487 10449520 PMC2195599

[B20] Jacobsen EA Ochkur SI Pero RS Taranova AG Protheroe CA Colbert DC Allergic pulmonary inflammation in mice is dependent on eosinophil-induced recruitment of effector T cells J Exp Med 2008 205 3 699 710 10.1084/jem.20071840 18316417 PMC2275390

