# Supplementary material for: Eosinophils affect functions of in vitro-activated human CD3-CD4+ T cells
Source: J Transl Med. 2013 May 6;11:112. doi: 10.1186/1479-5876-11-112 (PMC3659088; doi:10.1186/1479-5876-11-112)
Supplement: Additional file 1: Figure S1 — Eosinophil-induced enhancement of CD4 T cell proliferation is dose-dependent. Purified CD4+ T-cells from healthy subjects were activated with coated anti-CD3 and soluble anti-CD28 (1 μg/ml) antibodies for 48 hours, in absence and in presence of autologous IgA/anti-IgA activated eosinophils at various eosinophil-T cell ratios (0.1/1, 0.5/1, 1/1, and 2/1). Cells were then cultured for an additional 18 hours with H3-thymidine to assess proliferation. A statistically highly significant dose–response curve was observed when comparing T-cells alone to T-cells cultured with eosinophils at eosinophil/T cell ratios at 0.5/1 and 2/1 (Additional file 1: Figure S1a, n=7). A statistically significant progression was also observed when exploring additional ratios (Additional file 1: Figure S1b, n=4). [file 1479-5876-11-112-S1.pdf]

Supplementary Figure 2a. Eosinophil-induced enhancement of CD4 T cell proliferation is dose-dependent.

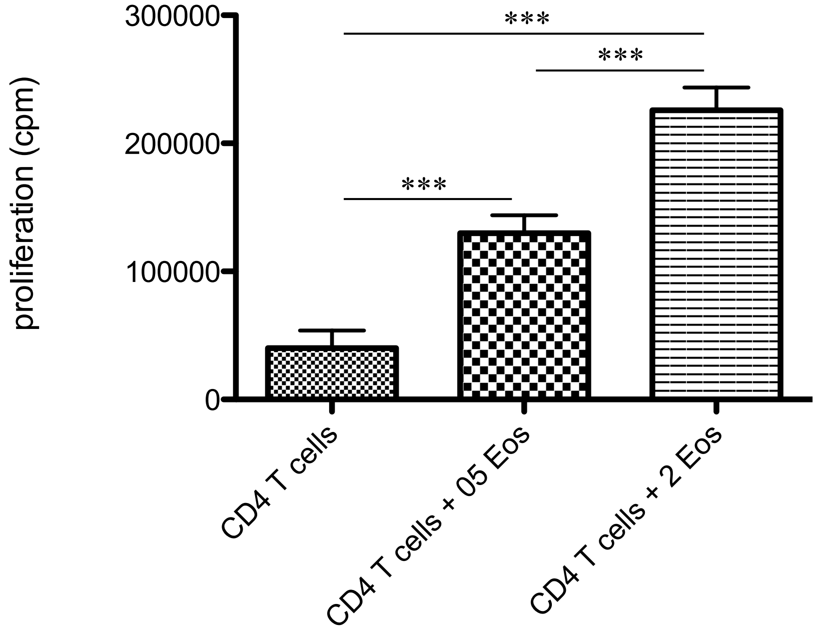

Supplementary Figure 2b. Eosinophil-induced enhancement of CD4 T cell proliferation is dose-dependent.

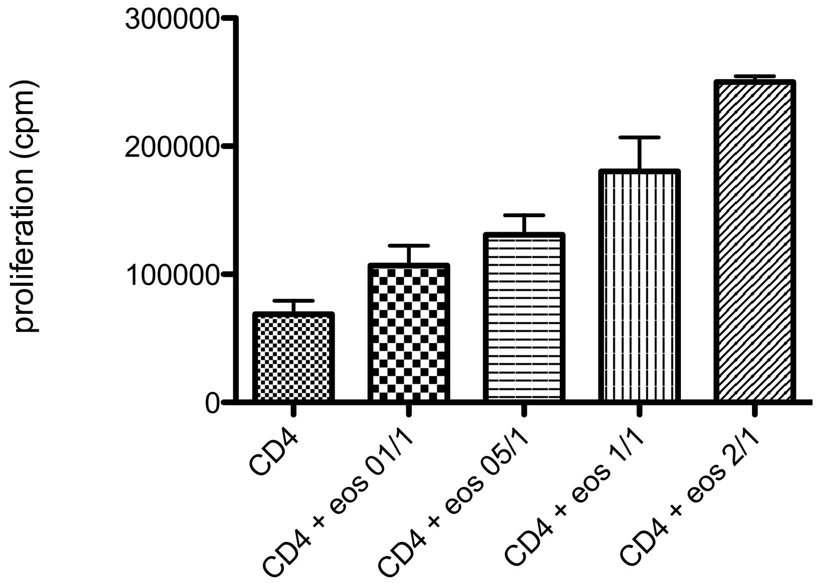

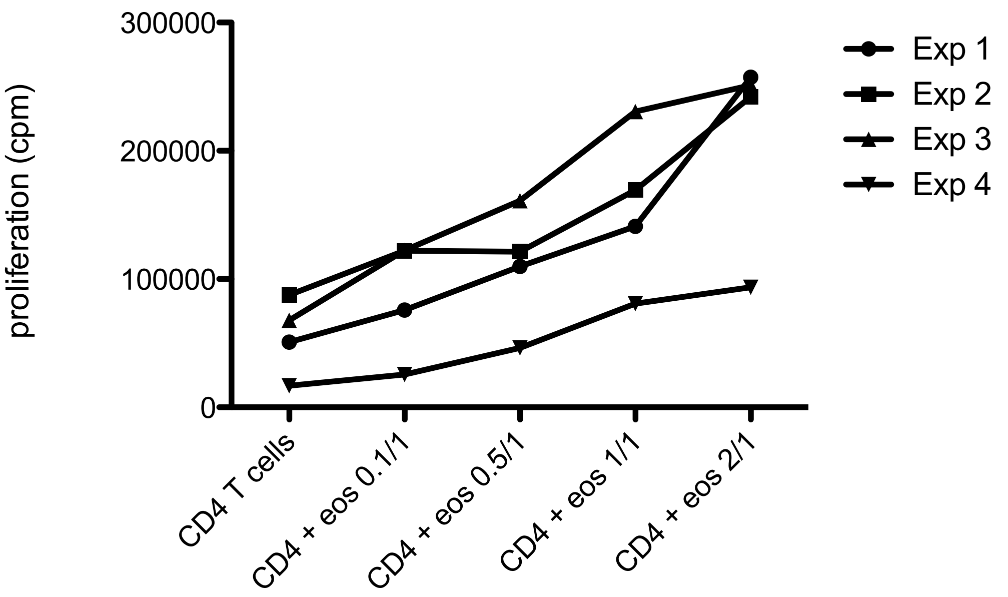

proliferation (cpm)
300000
200000
100000
0
CD4 T cells
CD4 + eos 0.1/1
CD4 + eos 0.5/1
CD4 + eos 1/1
CD4 + eos 2/1
Exp 1
Exp 2
Exp 3
Exp 4
