# Supplementary material for: Eosinophils affect functions of in vitro-activated human CD3-CD4+ T cells
Source: J Transl Med. 2013 May 6;11:112. doi: 10.1186/1479-5876-11-112 (PMC3659088; doi:10.1186/1479-5876-11-112)

Supplementary Figure 1.
Eosinophils decrease the percentage of $CD3^{-}CD4^{+}$ T-cells expressing IL-2 and IL-5.

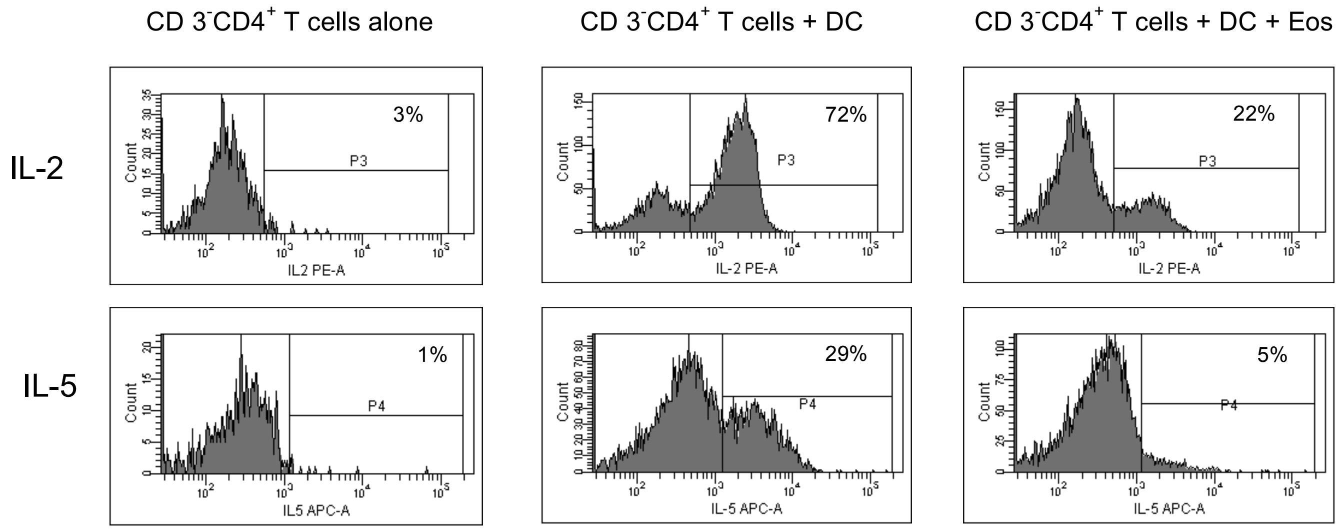

Supplement: Additional file 2: Figure S2 — Eosinophils decrease the percentage of CD3-CD4+ T-cells expressing IL-2 and IL-5. CD3-CD4+ T-cells from patient L-HES1 were cultured alone, in presence of LPS-matured dendritic cells, or in presence of both LPS-matured dendritic cells and eosinophils. On day 5, cells were harvested, washed, and re-stimulated with PMA+A23187 in presence of brefeldine A for a further 4 hours. Cells were then surface-stained with anti-CD3-FITC and anti-CD4-PerCP, fixed and permeabilised, then stained for intracytoplasmic cytokine expression with anti-IL-2-PE and anti-IL-5-APC. Acquisition for flow cytometry was performed on a Facs Canto II, using DIVA software. [file 1479-5876-11-112-S2.pdf]
